# Supplementary material for: HiGlass: web-based visual exploration and analysis of genome interaction maps
Source: Genome Biol. 2018 Aug 24;19:125. doi: 10.1186/s13059-018-1486-1 (PMC6109259; doi:10.1186/s13059-018-1486-1)
Supplement: Supplementary file 1 — Figures S1.–S7. Table S1. Supplementary material, and Supplementary references. (PDF 755 kb) [file 13059_2018_1486_MOESM1_ESM.pdf]

Supplementary Information

Supplementary Figures

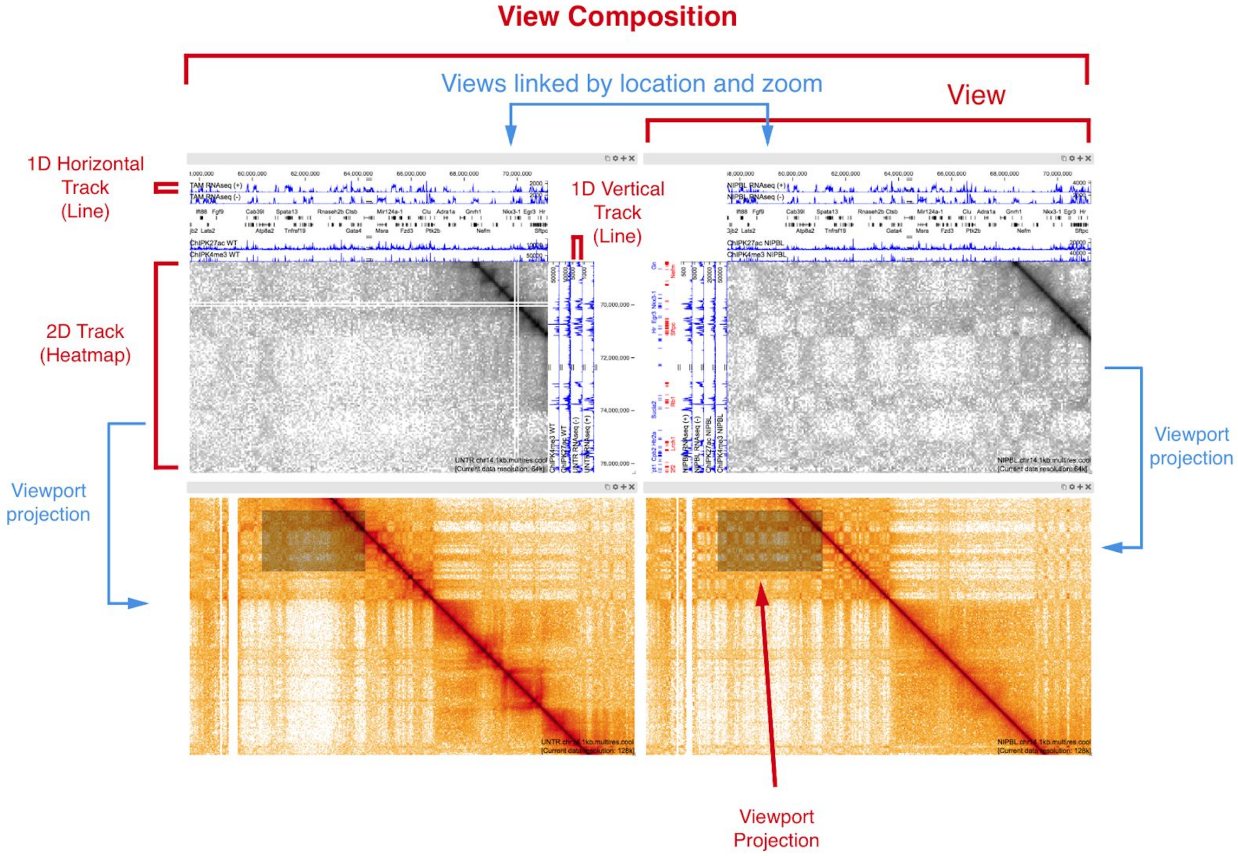

Figure S1 | Views and view composition. A view composition in HiGlass consists of a spatial arrangement of views, the data tracks within each view and the links that relate views between each other. In this figure, the top two views are linked by location and zoom so that whenever the user pans or zoom in one of them, the other automatically follows. This form of link only becomes noticeable with user interaction. Another type of link, the mirrored viewport, ensures that the visible area of one view is visible as an outline on another. In this example, the top two views mirror their viewports on the bottom two views.

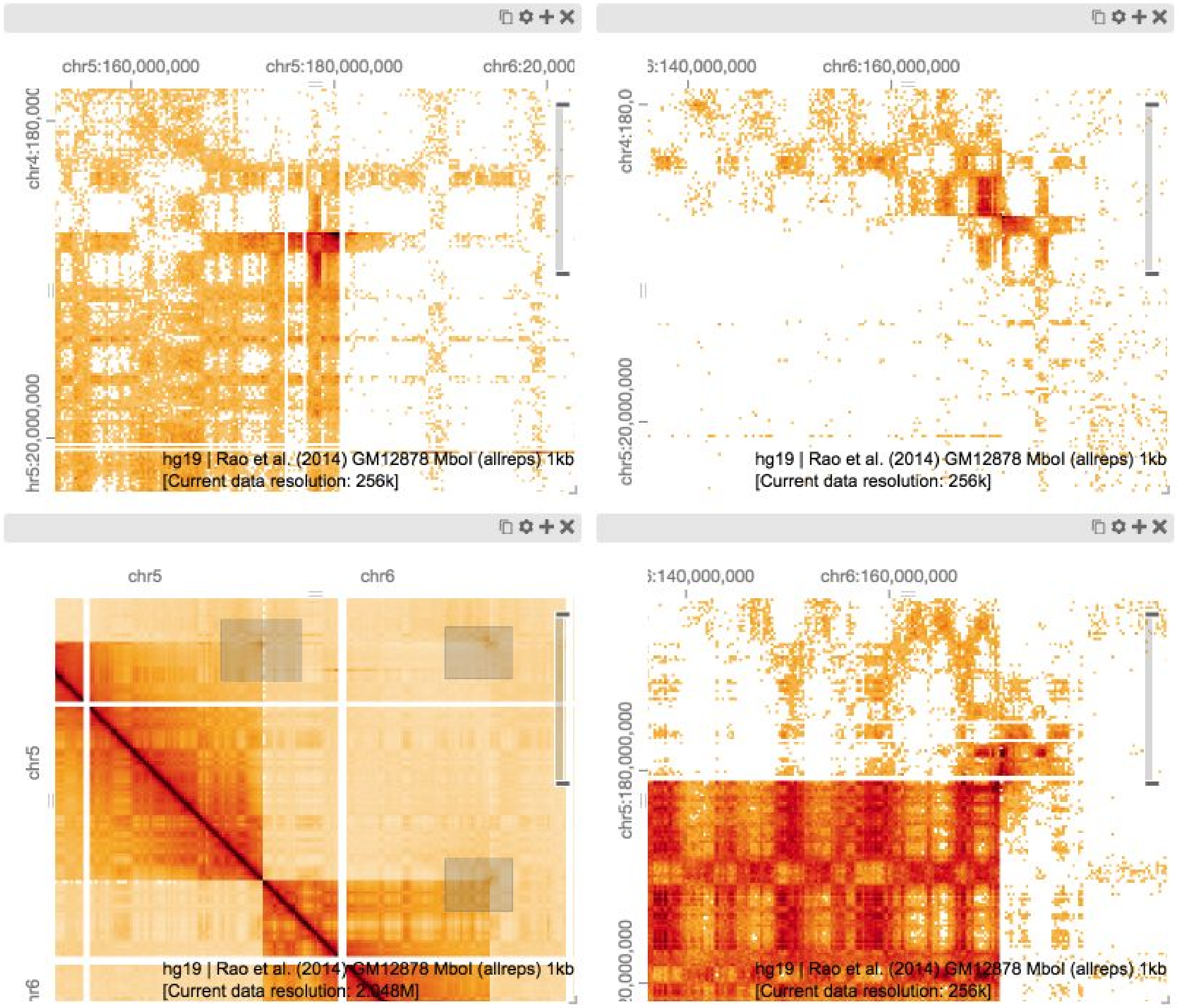

Figure S2: A view composition showing telomere clustering at the ends of chromosomes 5 and 6. (link: http://higlass.io/app/?config=Pfb7Bk1nTjWqsMnaJQy3FA)

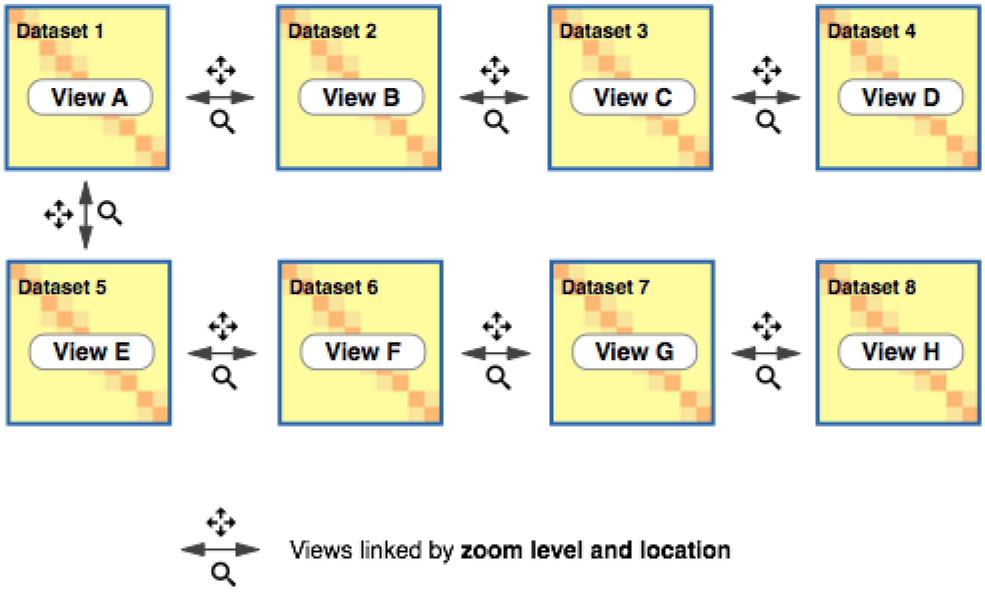

Figure S3 | Schematic of the 2D TAD comparison configuration. This schematic describes the linking present in Fig. 4. All eight views are linked by zoom and location, thus showing the same position at the same resolution.

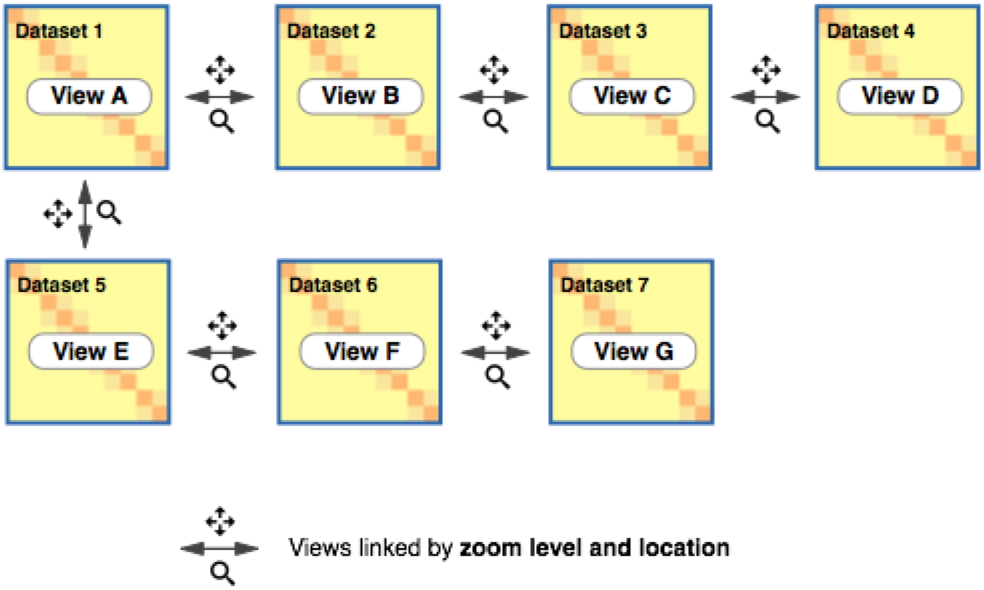

Figure S4 | Schematic of the 1D TAD comparison configuration. This schematic describes the linking present in Fig. 5. All seven views are linked by zoom and location, thus showing the same position at the same resolution.

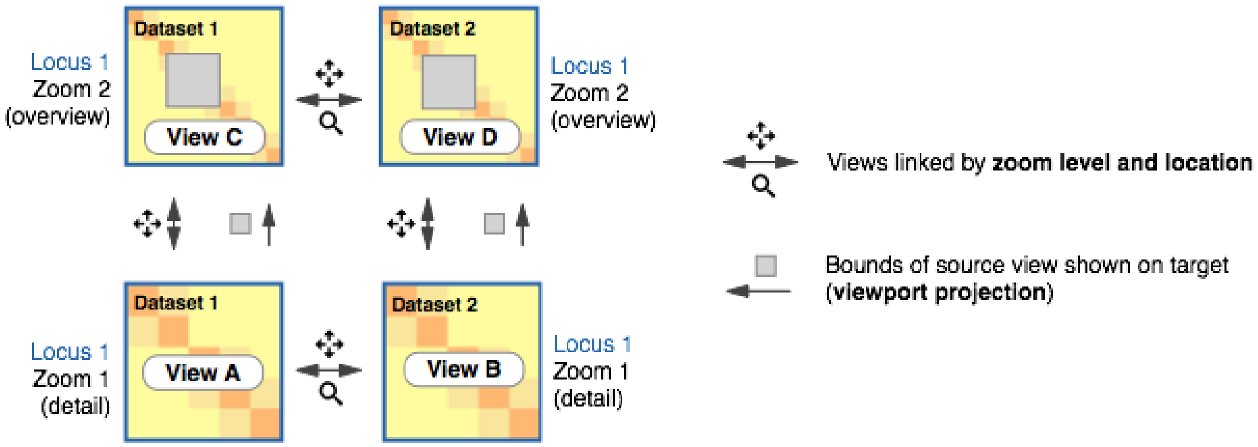

Figure S5 | Schematic of the two sample / two location / two zoom level comparison shown in Fig. 1. The two views on top always show the same location at the same zoom level, as do the two on the bottom. Because bottom views are usually (but not necessarily) more zoomed in than the top, the viewport projection shows their location in a subset of the region shown on top.

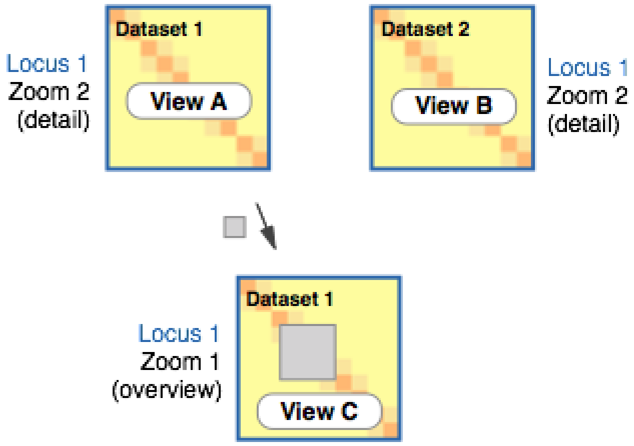

Figure S6 | Schematic of the exploratory view. This schematic describes the linking present in Fig. 2. The top two views are linked by zoom and location showing the same genomic position across two samples. The bottom view shows the locations of the upper two but is controlled independently (not linked).

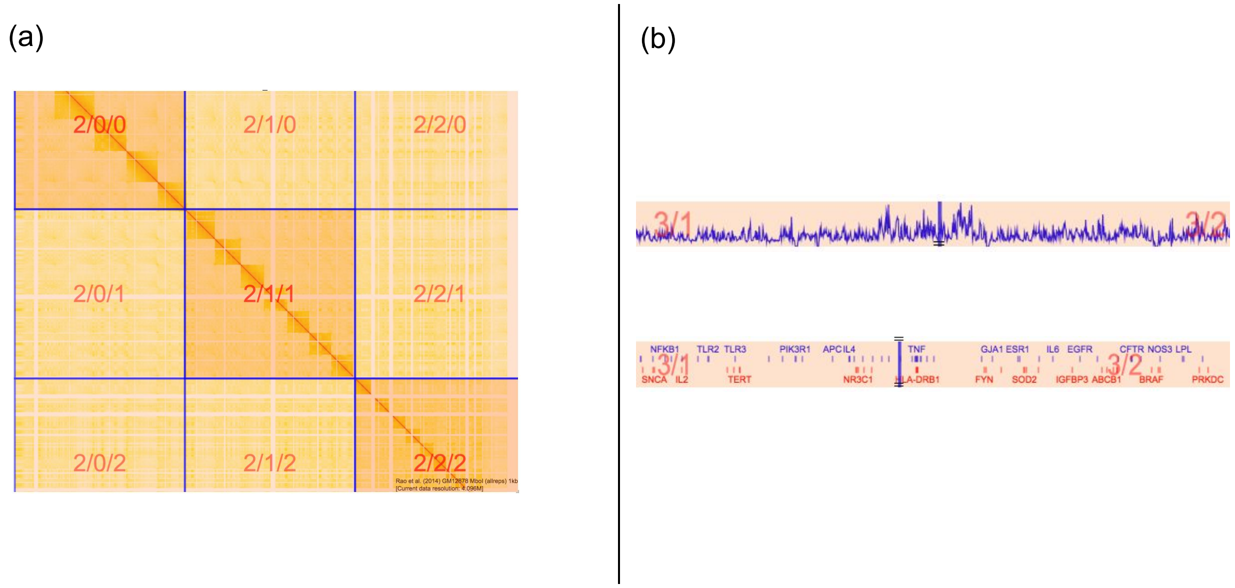

Figure S7 | (a) An example of the subdivision of a heatmap into tiles. The first digit corresponds to the zoom level while the second and third correspond to the x and y position of the tile. (b) Other types of tracks such as lines and gene annotations are similarly divided into tiles and loaded in a piecewise fashion. Because these data are displayed along one dimension, they only require a zoom level and x coordinate.

	Highest resolution size	Multi-res size	Size increase
Dixon et al. (2012) H1hESC HindIII (allreps) 1kb	864M	1.9G	2.2x
Zuin et al. (2014) HEK293Rad21Control HindIII (allreps) 1kb	551M	4.2G	7.6x
Jin et al. (2013) IMR90_Tnfa HindIII (allreps) 1kb	1.0G	8.1G	8.1x
Rao et al 2014, Mbo I (allreps) 1kb	3.5G	22G	6.3x

Table T1 | File size comparison for single and multiple resolution cooler files. Each multi-resolution cooler file contains resolutions that are multiples of 2 greater than 1K (e.g., 1K, 2K, 4K… 16M).

Supplementary Methods

View Composition and Sharing

The user interface for HiGlass is built to display multiple datasets and to facilitate their exploration through the synchronization of navigation. Supplementary Fig. 1 provides an overview of the terms we use to describe elements of the user interface. Broadly, views are individual units that can be navigated (zoomed and panned) independently or in concert with other views. Tracks are subsections of views that are always synchronized along one or two of their axes. Links describe relationships between the different views and serve to enforce synchronized navigation. Viewport mirroring displays the extent of one view in the coordinate system of another.

The view composition, or spatial arrangement and linking between views and tracks, can be configured using the user interface (https://github.com/hms-dbmi/higlass/wiki/View-operations) or defined in an external JSON file. Compositions created through the user interface can be exported as JSON configuration files to be shared and reproduced without having to move large data files. There is also an option to store a JSON configuration file on the server and access it through a hyperlink (https://github.com/hms-dbmi/higlass/wiki/Exporting-and-sharing).

HiGlass Architecture

HiGlass is composed of four components: the client, the server, webapp and the docker container (Fig. 6). This HiGlass client is responsible for data visualization. The higlass server provides data on demand and maintains a list of available data tracks. The higlass web application uses the higlass client to display data. Each of these components is largely independent and can be used independently. HiPiler, a tool for efficiently exploring multiple hundreds to thousands of loci simultaneously, uses the HiGlass client within its own separate web application (Lekschas et al. 2017). The HiGlass server can be deployed on its own and accessed by multiple HiGlass clients. Our public server, for example, provides access to multiple published datasets using an api accessible at http://higlass.io/api/v1.

For standalone use, we provide a Docker container which contains the latest version of the server, client and web application. For example, the Docker container can be used to start a server on the user's computer to view local files. It can also be configured to query a public server for available data and common tracks such as gene annotations or chromosome sizes. When associated with a public IP, a running HiGlass Docker can serve as a fully functioning,

publicly accessible version of HiGlass. When used within a private network, it can be used to visualize private data within a controlled environment.

Data Processing

Schwarzer et al. 2017

The Hi-C data used to create Figures 2 and 3 of this study was generated as described in Schwarzer et al. (Schwarzer et al. 2017) Hi-C data was binned using the *cooler* Python package (https://github.com/mirnylab/cooler) to produce binary Cooler (.cool) files. The Cooler format is a realization of a sparse matrix storage schema for genomic interaction matrices implemented using HDF5 (HDF5 Group 1997-2017), a scientific data container format that supports features including chunking, compression and random array access. The details of the Cooler format and *cooler* package are described at http://cooler.readthedocs.io.

Starting with .cool files containing single Hi-C matrices binned at 1 kb resolution, we use the `zoomify` command from the *cooler* package, which recursively rebins the chromosomal blocks in 2x2 pixel groupings to generate multi-Cooler files containing contact matrices of several resolutions, starting from the input matrix (at the "base" resolution) up until a maximally coarsegrained matrix (the first aggregation to result in a genomewide matrix of 256 x 256 pixels or less). Each of the resulting contact matrices was normalized by computing a vector of balancing weights using a sparse implementation of the iterative correction or matrix balancing approach (Imakaev et al. 2012), which is stored as part of the genomic bin table of the corresponding contact matrix in the file.

The ChIPSeq and RNASeq tracks were converted to `hitile` format for loading into HiGlass using the clodius (http://github.com/hms-dbmi/clodius) package. We use this HDF5-based format in order to obtain fast random-access at any location within the genome.

The resulting multiresolution files are loaded onto the higlass server as described in the higlass documentation (http://github.com/hms-dbmi/higlass/wiki).

Forcato et al. 2017

The TAD calls used in Figures 4 and 5 were obtained from the data in Forcato et al. 2017 (Forcato et al. 2017). The raw TAD calls were converted into the `bed2ddb` format which HiGlass uses to display 2D annotations using the clodius (http://github.com/hms-dbmi/clodius) package. This format is a defined schema for the sqlite database which supports fast random access for spatial queries.

Information about processing and importing data into higlass is available on the wiki:

https://github.com/hms-dbmi/higlass/wiki

Supplementary References

Forcato M, Nicoletti C, Pal K, Livi CM, Ferrari F, Bicciato S. 2017. Comparison of computational methods for Hi-C data analysis. *Nat Methods* **14**: 679–685.

Imakaev M, Fudenberg G, McCord RP, Naumova N, Goloborodko A, Lajoie BR, Dekker J, Mirny LA. 2012. Iterative correction of Hi-C data reveals hallmarks of chromosome organization. *Nat Methods* **9**: 999–1003.

Lekschas F, Bach B, Kerpedjiev P, Gehlenborg N, Pfister H. 2017. HiPiler: Visual Exploration of Large Genome Interaction Matrices with Interactive Small Multiples. *IEEE Trans Vis Comput Graph*. http://dx.doi.org/10.1109/TVCG.2017.2745978.

Schwarzer W, Abdennur N, Goloborodko A, Pekowska A, Fudenberg G, Loe-Mie Y, Fonseca NA, Huber W, Haering C, Mirny L, et al. 2017. Two independent modes of chromatin organization revealed by cohesin removal. *Nature*. http://dx.doi.org/10.1038/nature24281 (Accessed October 5, 2017).
